# Supplementary figures and images for: Nutrient stress dramatically increases malaria parasite clag2 copy number to increase host cell permeability and enable pathogen survival
Source: PLoS Pathog. 2025 Oct 16;21(10):e1013321. doi: 10.1371/journal.ppat.1013321 (PMC12530616; doi:10.1371/journal.ppat.1013321)

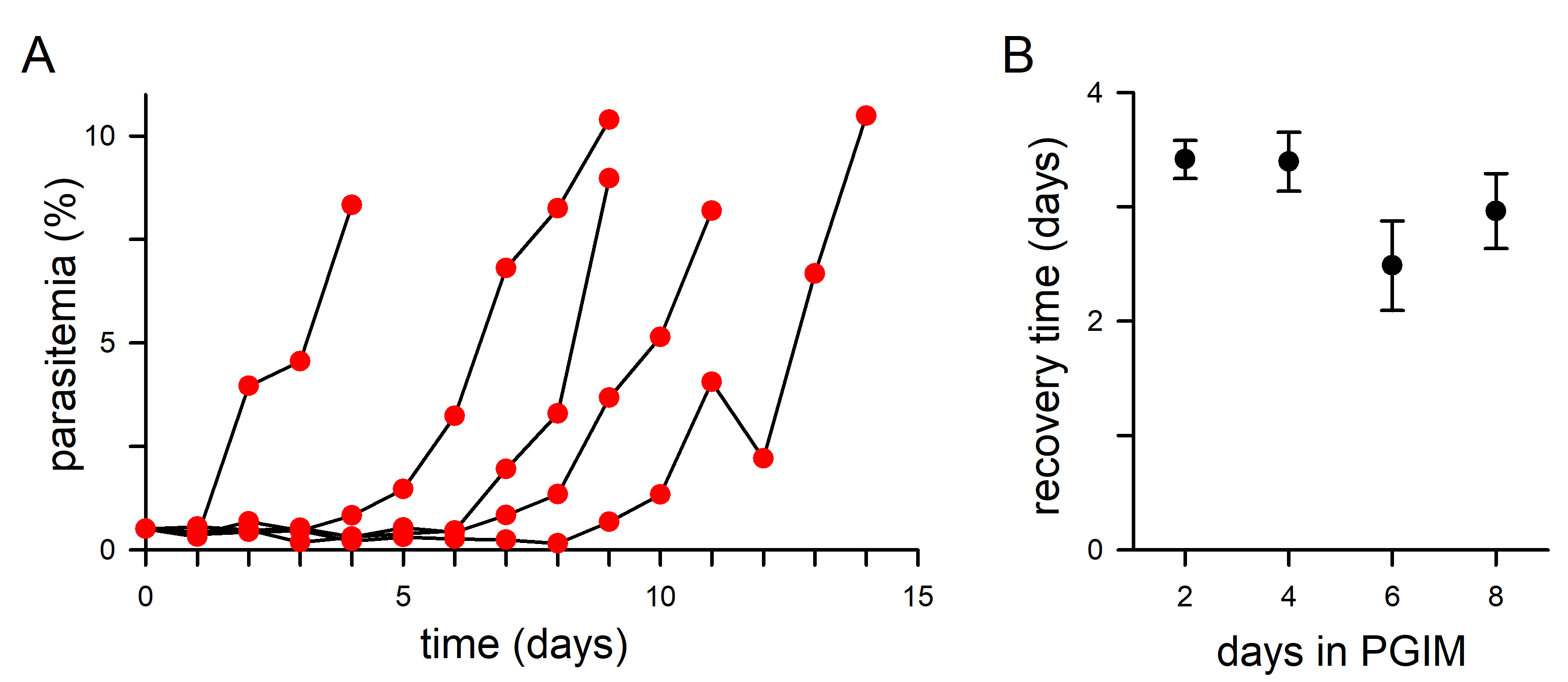

Supplement: S1 Fig — A) C3h-KO expansion after cultivation in PGIM for 0, 2, 4, 6, or 8 days prior to return to RPMI 1640-based medium (left to right curves; symbols represent mean of 3 trials). Increasing the duration of PGIM exposure does not extend the lag before resumed growth. B) Time to 2% parasitemia after return to RPMI 1640-based medium for C3h-KO cultures seeded in PGIM for indicated number of days. Symbols represent mean ± S.E.M. estimated by linear interpolation of daily parasitemia estimates from microscopic observation; n = 3 trials each. Recovery time does not increase with extended PGIM exposure, indicating that these parasites remain viable. (TIF) [file ppat.1013321.s001.TIF]

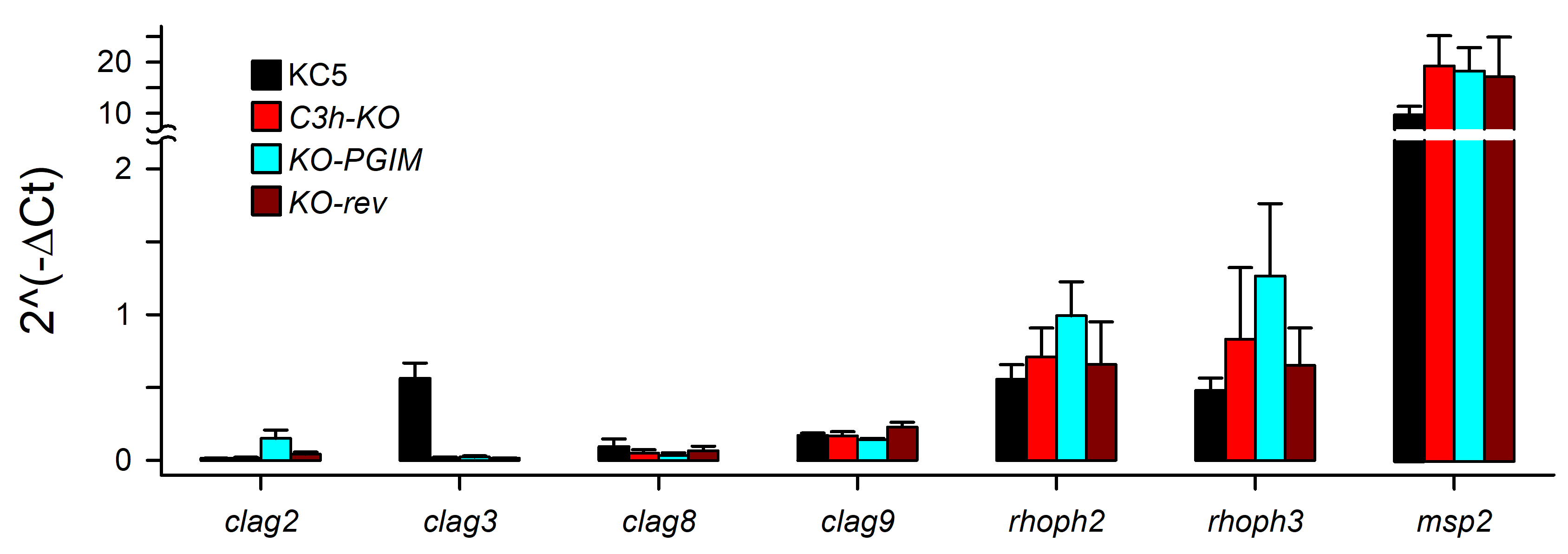

Supplement: S2 Fig — Mean ± S.E.M. transcription of indicated clag and rhoph genes in KC5, C3h-KO, KO-PGIM, and KO-rev parasites (black, red, cyan, and dark red bars in each group, respectively), calculated as 2(-ΔCt) using ΔCt relative to the α-tubulin loading control. msp2, unrelated gene with similar stage-specific expression. Values reflect the RT-qPCR experiments shown in Fig 5A without normalization to the wild-type control. (TIF) [file ppat.1013321.s002.TIF]

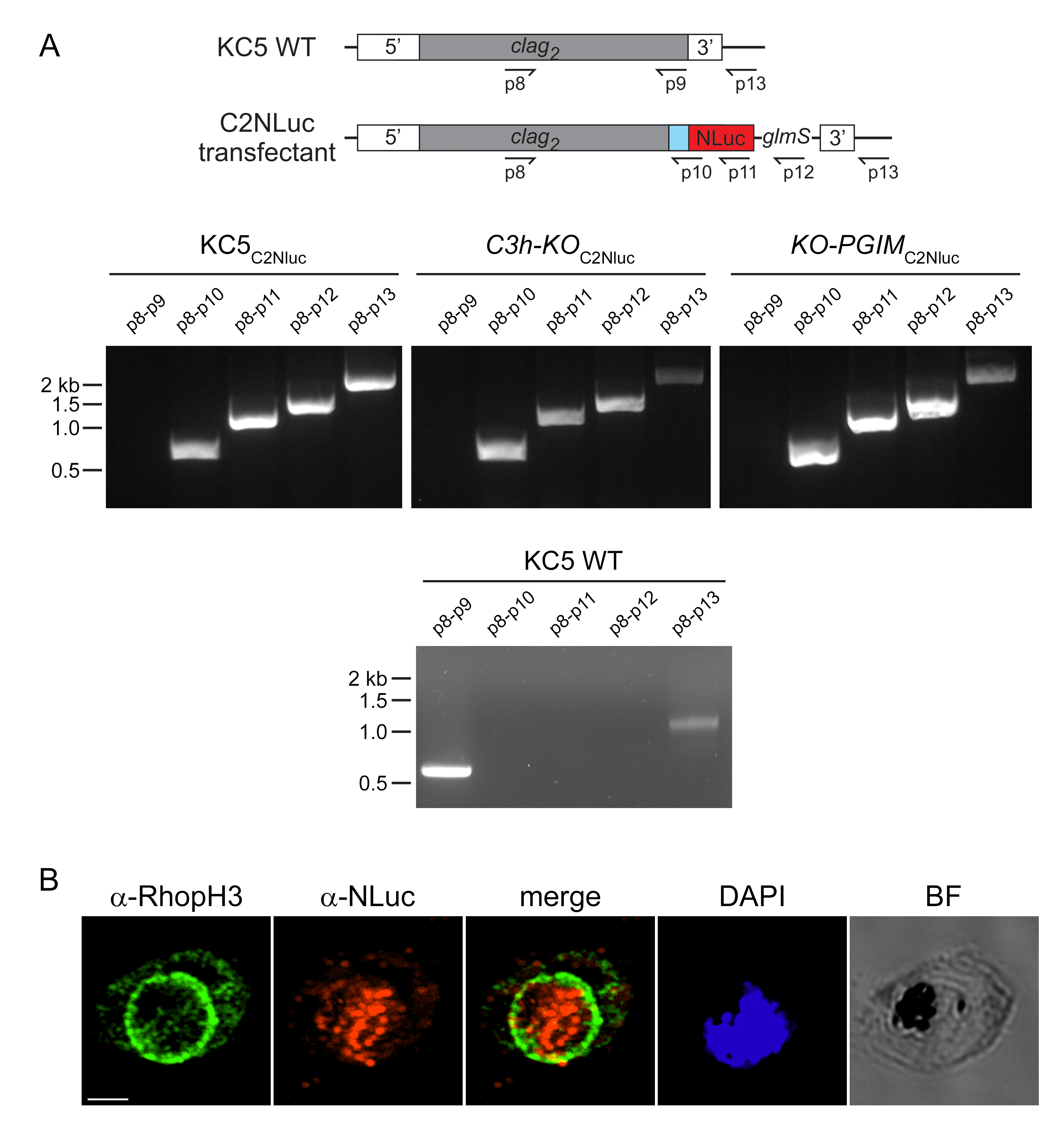

Supplement: S3 Fig — A) Ethidium-stained gels using indicated primers and DNA from transfectant clones and the wild-type KC5 parent. Primer pair p8-p9 is specific for the wild-type clag2 locus while other pairs yield amplicons upon integration. Loss of the p8-p9 amplicon in KC5C2NLuc, C3h-KOC2NLuc, and KO-PGIMC2NLuc indicates complete replacement of all genomic clag2 copies with the integration cassette, which adds a C-terminal NanoLuc reporter. Expected sizes (in bp): p8-p9, 605; p8-p10, 619; p8-p11, 1027; p8-p12, 1465; p8-p13, 1975 in transfectant lines and 1066 in wild-type KC5. Primer sequences are in S1 Table. B) Indirect immunofluorescence confocal microscopy images of a trophozoite-stage KO-PGIMC2NLuc parasite probed with anti-RhopH3 and anti-NanoLuc antibodies (green and red, respectively). Scale bar, 5 µm. The NanoLuc-tagged CLAG2 protein is delivered to infected cells after invasion and is exported into host cytosol [24]. (TIF) [file ppat.1013321.s003.tif]

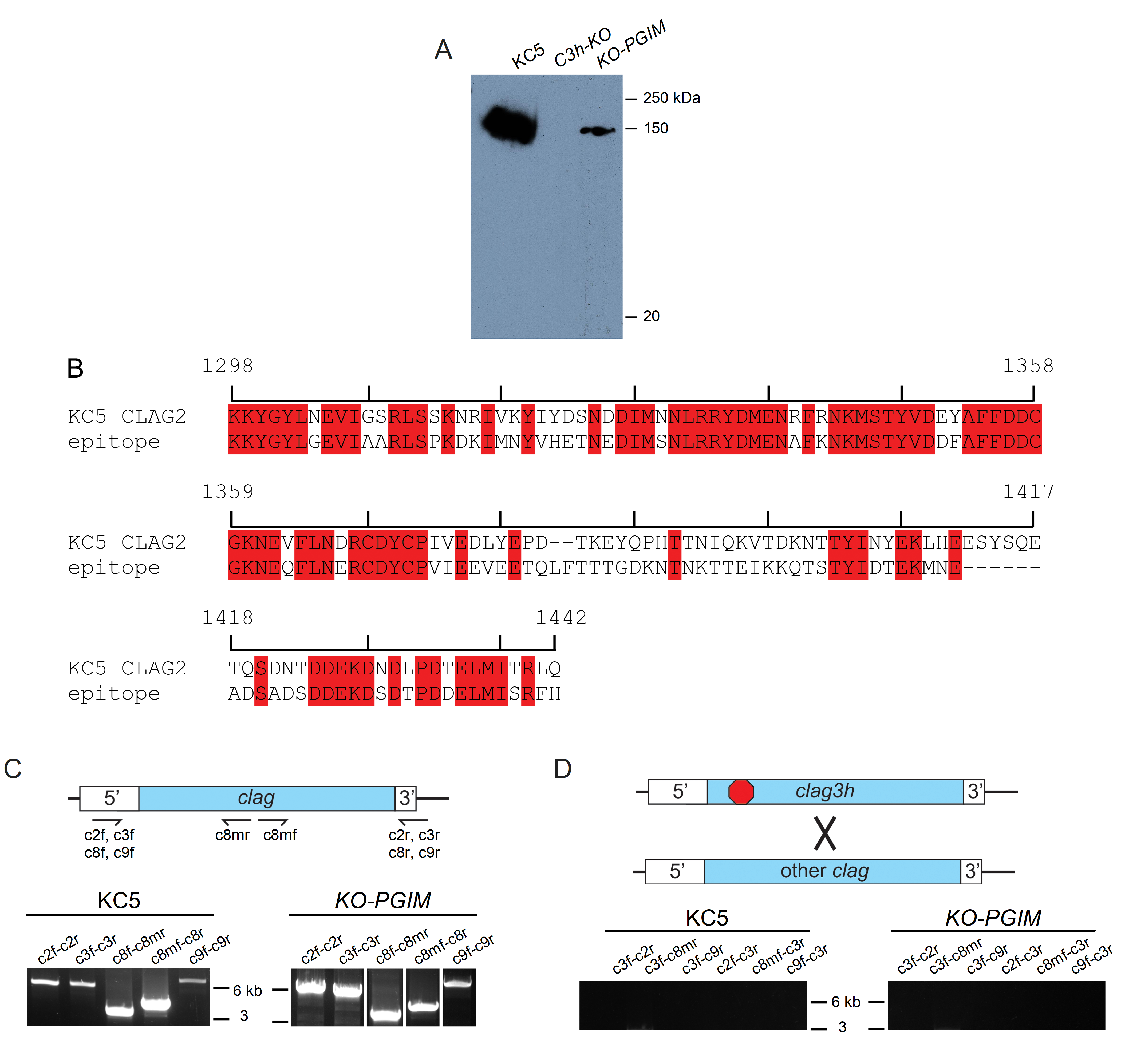

Supplement: S4 Fig — A) Anti-CLAG3 immunoblot using total membranes from indicated parasites. Samples used percoll-sorbitol enriched infected cells and were match-loaded. A low-intensity band is detected in KO-PGIM lysates but absent in the unselected C3h-KO parent. B) Sequence alignment of KC5 CLAG2 sequence with the recombinant epitope used to produce anti-CLAG3 antibody. Identical residues are highlighted in red; the ruler is numbered according to CLAG2. As the band detected in KO-PGIM reflects cross-reaction with the CLAG3 C-terminal epitope, we did not attempt to quantify band intensities. C) Ethidium-stained gels showing unaltered detection of clag genes in KO-PGIM when compared to the wild-type KC5 line. PCR of clag8 was performed in two fragments to facilitate specific detection. Ribbon schematic at top shows the sites recognized by each primer; primer sequences are in S1 Table. D) Gels showing absence of ectopic recombination between clag3 and other paralogs in either KC5 or KO-PGIM. (TIF) [file ppat.1013321.s004.TIF]
