# Supplementary material for: Nutrient stress dramatically increases malaria parasite clag2 copy number to increase host cell permeability and enable pathogen survival
Source: PLoS Pathog. 2025 Oct 16;21(10):e1013321. doi: 10.1371/journal.ppat.1013321 (PMC12530616; doi:10.1371/journal.ppat.1013321)
Supplement: Attachment — Submitted filename: Itemized Responses to Reviewers 092925.docx [file ppat.1013321.s008.docx]

**Itemized response to reviewer comments**

**Reviewer #1:**

*This manuscript presents compelling and well-executed work that challenges the prevailing view of PSAC composition by demonstrating that P. falciparum parasites lacking CLAG3 can restore solute uptake and growth under nutrient-limited conditions via amplification of the clag2 gene. The authors provide strong genetic and phenotypic evidence suggesting that CLAG2 can compensate for the loss of CLAG3, leading to a revised model of channel formation and nutrient acquisition in the parasite. This finding highlights the genomic plasticity of P. falciparum and extends our understanding of how the parasite adapts to environmental stress.*

We thank the reviewer for their positive comments.

*However, some conclusions—specifically those regarding isoleucine uptake via PSAC—require a more cautious interpretation. Isoleucine is a bulky, hydrophobic amino acid not generally considered a primary PSAC substrate. The observed reduction in isoleucine uptake in CLAG3 knockout lines may reflect indirect effects on parasite physiology or other transport systems (e.g., amino acid transporters such as PfAAT1 (PF3D7_0629500)) rather than direct passage through PSAC. Acknowledging this limitation and refining some interpretations would strengthen the manuscript. Additionally, clarification of the parasite stage at which permeability and expression assays were performed is important, particularly in the context of possible altered IDC duration under nutrient-limited conditions.*

We thank the reviewer for these questions. While it is correct that other transporters within infected cells may also transport isoleucine (e.g. *Pf*AAT1), these transporters are at intracellular membranes, not the host membrane (*Pf*AAT1 at the digestive vacuole membrane, PMID: 29326268 and 36976018). Because the osmotic lysis assay we used yields a lytic signal upon erythrocyte lysis, it **can only track only ion and solute fluxes at the host membrane.** Transport through other channels/carriers at intracellular membranes would not produce osmotic lysis as it does not create a gradient for water entry into the infected cell. This point applies to all permeant solutes such as sugar alcohols (e.g. sorbitol), amino acids (e.g. isoleucine and proline), etc. with increased host membrane permeability.

Work from various labs have shown that isoleucine uptake at the host membrane after infection is almost entirely via PSAC, so it is not correct that isoleucine is “not generally considered a primary PSAC substrate”. PMID:3887158 found that uninfected cells do not take up sufficient isoleucine to produce osmotic lysis and that *P. falciparum* infection produces a large increase in isoleucine permeability (their Table II); their paper correlated this finding with radioisotope uptake at the same membrane. PMID:17047158 confirmed this finding and linked it to linked it to a shared solute channel. PMID:17287402 shows negligible uptake in uninfected cells and linked isoleucine uptake to PSAC (their Fig. 1). We established PSAC as the primary molecular mechanism for isoleucine uptake at the host membrane with conditional knockdown of PSAC subunits (Fig. 4-Fig.Supp. 1 of PMID:28221136) and pharmacological studies (Figs. 1, 3, 4 of PMID:19050955).

*Part II – Major Issues:*

*No new experiments are strictly required for acceptance.*

*However, the following points should be addressed in the text to clarify interpretations and add context to the results:*

*1. Refine the interpretation of isoleucine as a PSAC substrate:*

*The manuscript currently suggests that isoleucine is acquired via PSAC (e.g., Page 4, lines 14–16). This statement should be revised or qualified, as current knowledge does not support isoleucine as a primary PSAC substrate due to its size and hydrophobicity. The authors should consider alternative explanations for the observed decrease in isoleucine uptake in CLAG3-deficient lines.*

As described above, multiple studies from several groups have established isoleucine is indeed a primary PSAC substrate and exclude significant contributions from other transporters. To clarify this important issue for readers, we have revised and expounded “Both solutes are primarily acquired … RhopH2 and RhopH3 [24].” p. 4, line 23 – p. 5, line 2.

*2. Clarify parasite stage and IDC timing under PGIM conditions:*

*According to Subudhi et al transcriptomic data (DOI: 10.1038/s41467-020-16593-y) deposited in PlasmoDB, clag3.1 and clag3.2 are expressed primarily between 34–48 hours post-invasion. Thus, since CLAG3 expression is stage-specific, it is essential to state whether parasite stages were matched across conditions. However, the experimental timepoints (in hours post-invasion) used for permeability assays and RNA/protein analyses are not clearly stated. Also, the PGIM medium could affect the parasite’s IDC, potentially prolonging the cycle, this could influence gene expression and protein levels. The authors should specify the time post-invasion at which experimental assays were performed, and whether parasite staging was carefully matched between conditions to ensure valid comparisons.*

This is also an important point. Yes, *clag* transcription and translation are limited to 34-46 h in the asexual cycle, with the best experimental evidence in PMID:15049814 (Fig. 7 showing Northern blotting of *clag, rhoph2, rhoph3* using stringently synchronized parasites). Remarkably, our work and that of others establish that PSAC activity is induced at an *earlier* point in the parasite cycle (fully induced without further increases in osmotic lysis kinetics or patch-clamp currents between 24-30 h post-invasion). Channel activation prior to gene transcription, while surprising, is possible because PSAC subunits are transcribed and translated in the preceding erythrocyte cycle, packaged into rhoptries as the RhopH complex (named for this initial site during transit), transferred to new erythrocytes upon invasion, and exported to the host membrane for channel formation many hours after transcription (see Fig. 8 of PMID:28221136).

As PSAC activity is fully induced by the early to mid-trophozoite stage, we have used synchronized trophozoite- and schizont-stage cultures for all transport studies, as stated in the Methods section entitled “Solute uptake measurements”, p. 18, now edited to clarify this point. Protein blots were performed using similarly matched cultures as described under Methods, “Protein blots”, p. 19. Quantitative RT-PCR for gene transcription studies used tightly synchronized, matched schizont-stage infected cells as this is the time of *clag* and *rhoph* gene transcription. To achieve consistent results, we used serial smears and described this in Methods, “Quantitative reverse transcriptase PCR”, p. 21. As transcription of these genes occurs at the early schizont stage, we added *msp2* as a second control to ensure schizont stage-specific matching for RT-PCR studies.

Although our prior studies with PGIM did not reveal measurable changes in the duration of the parasite cycle, we agree that use of altered media can influence both gene expression and cellular phenotypes. Use of synchronous trophozoite-stage cultures for transport assays and the *msp2* stage-specific control in addition to the constitutive *α-tubulin* control for RT-PCR studies addresses this concern and ensures valid comparisons.

We have clarified that transcription is temporally separated from induction of channels, which are induced in the subsequent cycle. See new paragraph to start the Results, “CLAG proteins … early trophozoite stage [24].” p. 4, lines 13-18.

*Part III – Minor Issues:*

*1.Wording revisions:*

*Revise sentence on Page 4, lines 14–16 to something more cautious, e.g.:*

*"We examined sorbitol and isoleucine permeabilities in C3h-KO and KC5; while sorbitol is a well-established PSAC substrate, isoleucine uptake may involve additional or alternative transport pathways."*

Multiple studies described above show that isoleucine uptake occurs primarily via PSAC at the host membrane; the revisions described above address this important point.

*2.Suggestion for future experiments (optional, in discussion) to better understand the link between isoleucine uptake and direct PSAC-mediated transport, the authors could consider:*

*- Performing competition assays using sorbitol or other known PSAC substrates to determine if isoleucine uptake is competitively inhibited.*

Our studies suggest that the PSAC pore only weakly interacts with permeating solutes and that it therefore does not saturate until concentrations are > 500 mM (Fig. 7 of PMID:15319279). Such nonsaturating pores generally do not exhibit measurable competitive inhibition between solutes. As molecular studies already indicate that these substrates share a common pore (CLAG3 knockout <PMID:32069335> and *rhoph* conditional knockdowns <PMID:28221136>), it is not clear what new insights could be gained by competition studies.

*- Conducting transcriptomic analysis comparing WT and CLAG3-KO lines, with a focus on amino acid transporter expression.*

This would be interesting if reduced host membrane uptake through CLAG3 knockout produced compensatory changes in intracellular nutrient transporter gene products. It would be outside the scope of studying exported proteins and host membrane permeability changes as we envisioned with the present study. We agree with this comment and have included “In addition to changes in PSAC-associated … parasite digestive vacuole [63].” Discussion, p. 16, line 21 – p. 17, line 2.

*- Generating and testing a PfAAT1 KO or knockdown strain in the clag3-KO background to assess compensatory or synergistic effects on isoleucine uptake.*

As discussed above, *Pf*AAT1 localizes to the digestive vacuole membrane (PMID: 29326268 and 36976018) and does not contribute to host membrane permeability or isoleucine uptake from plasma. We agree that there might be interesting changes in intracellular membrane transport properties that compensate for reduced host membrane permeability in the CLAG3 knockout. We have added a statement about *Pf*AAT1 to address this issue, Discussion, p. 16, line 21 – p. 17, line 2.

*3. Address clag2 pre-existence (Figure 4B):*

*The observed clag2 gene amplification under amino acid-limited conditions is a striking example of adaptive genome remodeling. As the authors note, this supports a broader model where nutrient stress, like drug pressure, can drive copy number variations to enhance parasite survival. This aligns with known mechanisms for resistance involving pfmdr1 or pfpm2/3. However, in Figure 4B, clag2 amplification may appear detectable in the C3h-KO parental line, (although not confirmed by WGS) suggesting that some degree of clag2 amplification may have pre-existed in the population, raising the possibility of selection on a pre-existing subpopulation rather than de novo amplification. The authors should discuss whether low-level pre-existing amplification may have been selected under PGIM pressure, or whether this reflects background noise. This clarification will help support the model of clag2-driven adaptation.*

Figure 4B indeed reveals some *clag2* amplification. As with drug selections, one can wonder whether such a subpopulation was present before production of the *clag3* knockout line. All we know is that, if present, it was below the detection threshold in the PCR strategy used in Fig. 4B. The consensus view is that mutations, indels, and CNV arise with some regularity in culture but disappear quickly unless there is an associated survival advantage. CLAG2 duplication presumably arises regularly in culture but disappears because of fitness cost. CLAG3 knockout reduced host membrane nutrient uptake and appears to have shifted the balance of the forces governing *clag2* copy number, permitting retention of a subpopulation with CLAG2 amplification despite growth in nutrient rich RPMI 1640-based medium. Greater selective pressure upon transfer to PGIM presumably shifted the balance yet further and enabled growth of individual parasites with larger scale amplification of the *clag2* locus. We believe the Discussion paragraph beginning with “We now report copy number variation …” covers these points with clear examples (p. 14). To further highlight the question raised here, we have added “As with mutations and indels, … larger scale *clag2* amplifications.” p. 15, lines 15-20.

*4. Figure S2A clarification:*

*In Figure S2A, the agarose gel analysis supports successful genome editing. However, assuming the primer pairs match those illustrated in Figure 5B, the p8 + p13 combination should, in principle, yield a smaller band in the KC5 line (wild-type for that locus). If this band is absent, please clarify whether this was expected or provide an explanation for the observed result.*

Agreed, *p8* + *p13* should produce a smaller amplicon with the wild type KC5 parasite, a good catch by this reviewer! We have rerun the PCR, obtained the expected amplicon with KC5 template, and replaced the graphic (now Fig. S3A in the revised manuscript). The missed amplicon in the prior trial presumably reflected a technical issue with the PCR.

*5. Minor formatting consistency:*

*Ensure that acronyms (PGIM) are defined at first use.*

Added. p. 5, line 16-17.

**Reviewer #2:**

*Summary:*

*The manuscript by Potchen et al. is a continuation of previous work from the group, in which they showed that CLAG3 proteins are an essential component of the parasite-encoded PSAC channel, involved in nutrient transport, and later demonstrated that CLAG3 is not essential for parasite growth in medium with supraphysiological nutrient concentrations (i.e., RPMI) but is essential for growth in medium with limited concentration of specific nutrients (PGIM). They also previously suggested (but not conclusively demonstrated) that other CLAG paralogs are also involved in PSAC formation.*

*Here the authors show that adaptation of a CLAG3-defficient P. falciparum line to grow in PGIM was associated with a compensatory amplification of the clag2 locus. This implies that CLAG2 can compensate for absence of CLAG3, providing evidence for the involvement of CLAG2 in PSAC formation. The results presented also demonstrate that CLAG2 and CLAG3 form channels with different characteristics (e.g., in terms of solute specificity).*

*The main conclusions are convincing and well demonstrated by the data. The manuscript is technically sound and clearly written, and the figures are also clearly presented. I only have minor suggestions to improve the manuscript, which do not require new experiments.*

We thank this reviewer for their accurate summary and positive comments about our manuscript.

*Part II – Major Issues:*

*-*

*Part III – Minor Issues:*

*It is surprising that the copy number of clag2 increased up to 18-fold. Why are 18 copies of clag2 needed to compensate deletion of one copy of clag3? This massive increase may suggest that the CLAG2-based channel is less efficient than the CLAG3-based channel for the acquisition of the nutrients that are limiting in PGIM. An alternative possibility is that clag2 has a weaker promoter than clag3, which would imply that more copies of clag2 are needed to achieve the same transcript or protein levels. The latter possibility is supported by RNA-seq datasets available in PlasmoDB and several published RT-qPCR analyses. I recommend that the authors discuss these possible reasons for the unexpectedly high level of genetic amplification of clag2.*

Yes, these are possible explanations but there are several others including: differing affinities for interaction and transport in with required subunits (RhopH2 and RhopH3), differing efficiency of delivery and insertion at the host membrane, differing subunit stoichiometries of subunits in functional vs. nonfunctional channels, and/or differences in selectivity of the resulting channels. (The reviewer presumably was referring to differing channel selectivity when stating “less efficient than the CLAG3-based channel for the acquisition”.) Of note, regulated delivery of ion channel proteins to the plasma membrane is well-known, with delivery and assembly of AChR channel subunits having a maximum of 20-35% when experimental conditions are optimized; second messengers are also involved in regulating this process (PMID: 1849906). We have added “There are many … structure is solved”, p. 15, lines 7-14.

*-In fig. 5A, data is presented in a way that does not enable comparison of clag3 transcript levels in the parental line with clag2 transcript levels in the KO, KO-PGIM and KO-rev lines (only values for each transcript relative to the parental line are shown). Please provide a figure (a new panel in Fig. 5 or a new supplementary figure) that enables this comparison, showing the transcript levels (relative to tubuline) for each clag gene in each parasite line. It would be interesting to see if total clag transcript levels increase in the KO-PGIM line or, in spite of massive clag2 amplification, total clag transcripts remain more or less constant, because of the weaker promoter of clag2.*

We have added a supplementary figure (S2 Fig) showing these transcript abundances without normalization, which were calculated as 2^(-ΔCt)^ using ΔCt given relative to the α-tubulin loading control. The graphic shows that *clag3* has the highest transcript abundance of any *clag* in the wild-type parent and that *clag2* amplification only partially restores total *clag* abundance in *KO-PGIM*.

*-For a paper focused on increased clag2 expression as an adaptive mechanism, it would be relevant to discuss that clag2 was previously described as a clonally variant gene regulated by heterochromatin (at the epigenetic level), similar to clag3.1 and clag3.2 but unlike clag8 and clag9. This was demonstrated by transcriptional analysis of subclones that do or do not express the gene, and by genome-wide analysis of heterochromatin (a trait associated with clonally variant gene expression) showing that this epigenetic mark is present in clag2, clag3.1 and clag3.2 but not in clag8 or clag9. Clonally variant expression implies that the expression of this gene is dynamic and switching off the gene can provide a selective advantage under specific conditions, which fits with the observation that copy number decreased in the KO-rev line.*

We agree and have added this observation. “Although not subject … in *P. falciparum* [12,15].” p. 3, lines 21-23.

*-Some statements in the manuscript may suggest that copy number variation is a usual clag genes regulation mechanism that the parasite can use in natural infections to adapt to changes in the host environment (e.g., a malnourished host). This is missleading. Copy number variation was observed in an artificial parasite line with no functional clag3 gene, a situation that does not occur in the field (no natural isolates without functional clag3 genes have so far been reported, to the best of my knowledge). Furthermore, large copy number variation has not been described in field isolates. It is also important to note that copy number variation cannot provide the rapid and dynamic plasticity needed for adaptation to the changing conditions of the human host. Therefore, in natural infections, regulation of clag3 genes most likely relies on epigenetic mechanisms, which are much faster and dynamic than genetic changes. Studying the adaptive alterations in the CLAG3-defficient line to grow in PGIM was instrumental to establish the role of CLAG2 in the formation of PSAC, but statements that misleadingly suggest that clags copy number variation may be a common mechanism of parasite adaptation should be modified (mainly end of Abstract and Author Summary, and second paragraph of the Discussion: p.13 line 10 and elsewhere).*

We agree only partially with these statements. Certainly, epigenetic mechanisms allow for more rapid and reversible changes in adaptation to changes in the bloodstream environment (e.g. nutritional status, immunity, drug pressure). At the same time, our study establishes copy number variation can lead to adaptive changes, e.g. during host migration into environments or climates with altered nutrient availability. As an example, we point out that there are indeed large copy number variations for *clags* between *Plasmodium spp.*, ranging from as few as 2 in *P. berghei* and *P. knowlesi* to over 35 copies in *P. blacklocki*. Even within a single species, *P. falciparum* isolates are also known to have variable *clag* copy number without engineered *clag3* knockout as ref. 60 identified clones having between 3 and 7 *clag* copies.

Based on our findings, it appears likely that changes in *clag* gene copy number may have happened many times in *Plasmodium* evolution in response to nutrient availability, either during entry into new host species or during marked changes in nutrient availability. With sufficient time, new *clag* copies could then accrue mutations that further refine channel properties such as selectivity for specific nutrients. To clarify this point, we have made multiple revisions. See: “Our findings suggest that malaria parasites can change their DNA in response to sustained changes … into new host species.” p. 2, lines 8-10. “Although CNV generally cannot match … sustained changes in environment.” p. 14, lines 11-13. “When compared to other multigene copies, … range between 3 and 7 even without engineered *clag3* knockout [60].”, p. 15, line 22 – p. 16, line 4. “As with mutations and indels, … capacity for CNV under appropriate and sustained selective pressure.” p. 15, lines 15-21.

*-The results described in page 6 (Fig. 1E-G) indicate that the moderate decrease in permeability for the solutes tested in the KO line cannot account for the large growth defect of this parasite line in PGIM. The results presented afterwards do not provide an explanation for this intriguing observation, as increased CLAG2 levels restore permeability and largely revert the severe growth defect. Do the authors have an explanation, even if it is speculative, for why a small reduction in permeability in the KO line results in a severe growth defect? (larger than the effect on growth of a drug with a comparable impact on permeability). This should be discussed (in the Discussion).*

Our studies with the specific PSAC inhibitor ISG-21 revealed that the reductions in sorbitol and isoleucine permeabilities in the *C3h-KO* line are not sufficient to account for failure to propagate in PGIM. We believe our findings on this issue are well summarized by the two paragraphs: “The marked reduction in growth … failure to expand in PGIM.” p.6, line 14 – p. 7, line 6. To address this question, we have added “These findings are consistent … which remain unknown.” p. 7, lines 6-9.

*-End of page 9 and elsewhere. Why do the authors call this observation “unexpected”? If the gene encoding CLAG3 has been disrupted, it is expected that a channel with the properties of a CLAG3-based PSAC cannot be present in the cells.*

Two things are unexpected. First, despite disrupting CLAG3, a channel with properties of a CLAG3-associated PSAC is still present, as evidenced by permeability to organic solutes, comparable whole-cell currents with voltage-dependence and voltage-dependent inactivation, and similar pharmacology. Second, although the channel is still present, there are some surprising or unexpected changes in the channel’s biochemical properties: 1) complete refractoriness to external pronase E treatment, 2) retention of whole-cell Cl^-^ conductance despite loss of CLAG3 and marked increase above wild-type values in the *KO-PGIM* line, 3) unchanged voltage-dependence and voltage-dependent inactivation despite loss of the presumed pore-lining CLAG3 protein. This collection of observations is quite unexpected to us and challenges prevailing models of the channel structure.

*-Please indicated the position of the predicted TSSs for clag2 relative to the genetic amplification in Fig. 4A, using a zoomed-in view of the locus. The TSSs for most genes are described in several genome-wide datasets and available in PlasmoDB. This would give an idea of which is the functional unit amplified, presumably containing the full clag2 promoter.*

This is a good suggestion. The transcription start site for *clag2* is at chromosomal position 838594 in the latest version of *PlasmoDB*, some 750 bp downstream from the amplification event in our PacBio sequencing dataset (position 837841, as included in S2 Table). The amplified region runs to 844653 in our PacBio sequencing, quite near the end of the mRNA transcript’s end at 844668 according to *PlasmoDB*. We have added these values to the Fig. 4A legend as the small differences between mRNA start/end and genomic amplification start/end cannot be properly illustrated graphically for this large transcript. “The amplified region … in S2 Table.” p. 35, lines 1-3.

*-End of page 14. The data presented strongly suggest that different CLAGs confer different specificity and transport properties to the PSAC channel. Ref. 16 does not disprove this possibility, because it is possible that differences in nutrient permeability associated with different clag paralogs occur only for specific nutrients, which was not tested in ref 16. This should be made clear.*

We agree that one possible explanation for epigenetic regulation allows for fine-tuning of nutrient uptake and believe the language in this paragraph is fair to both views. We agree that failure to detect nutrient permeability differences upon switching is not proof of no difference, but there is no set of experiments that could formally “disprove” this possibility.

**Reviewer #3:**

*Summary:*

*PSAC is a parasite-induced nutrient channel in the RBC membrane important for parasite growth under physiological conditions. CLAG3 is key component of PSAC and this study shows that loss of CLAG3 expression arrests parasite growth in physiological media.*

*Loss of CLAG3 reduced Ile transport 3-4 fold but did not abolish it, indicating that transport is not fully dependent on CLAG3.*

*Long-term maintenance of clag3-deficient parasites in physiological media can select for revertant mutants that recover the ability to grow in physiological media and the transport rate of Sorbitol/Ile is restored to parental levels.*

*Changes in the relative transport of sorbitol vs Ile make it clear that the channel composition must be changing.*

*This reversion phenotype is associated with an 18-fold increase in the copy numper of clag2.*

*Subsequent growth of the revertants in RPMI leads to a reduction from 18 to 9 clag2 copies.*

*Change in copy number correlates well with expression change at mRNA level.*

*Tagging of the clag2 copies with NLuc showed ~3x increased protein levels in revertant relative to the C3h-KO parent but also shows ~4x increase in C3h-KO relative to KC5 parents with the intact C3h gene.*

*This is a well designed and executed study that sheds new light on how parasites can acquire nutrients and the relative functions of clag2 and clag3.*

We thank the reviewer for their detailed summary and positive comments.

*Part II – Major Issues:*

*1. While the correlation between increased clag2 and growth is strong, the authors would ideally directly effect the role of clag2 expression level on growth/transport modulating expression via the glmS element introduced during the Nluc tagging (as long as glucosamine transport does not depend on PSAC).*

*The standard approach using Blasticidin-selectable plasmids may be complicated if there is a major change in blasticidin transport, which would need to be tested.*

Our original design for the NLuc tag aimed to evaluate conditional knockout of *clag2* using the *glmS* riboswitch. Unfortunately, knockdown with glucosamine addition was suboptimal and variable, presumably due to the presence of multiple gene copies driven by separate promoters. Glucosamine toxicity on cultures also proved problematic in our attempts, as recognized by various *P. falciparum* studies using *glmS.* We have added “Although the transfection … toxicity on parasite cultures.” p. 13, lines 4-9.

The blasticidin S selection marker was required for this transfection because the prior transfection to produce *C3h-KO* utilized both other selection markers commonly used in *P. falciparum.* Fortunately, our prior studies with blasticidin S-resistant PSAC mutants established that the epigenetic silencing of *clag3* and *clag2* (both of which are required in our hands) only occurs in certain lines (not KC5 , as used here) and is fully reversible upon removal of blasticidin S. We have added “all transfectant lines without further exposure to blasticidin S.” to the Methods section to clarify this point. p. 23, lines 2-3.

*2. The mutational signature of the copy numbers suggest that amplification might have occurred in two steps, where the parental copy was amplified 8-10 fold, followed the acquisition of additional mutations one one of these copies, which was them amplified again. This is most apparent in the 3'UTR. This could be a sign that the additional mutations in clag2 are adaptive to growth in PGIM and leading to differential transport activities of the two sets of clag2 copies. Do any of these mutations result in coding changes? If not, this can be disregarded.*

S3 Table lists all mutations in KC5, *C3h-KO*, and *KO-PGIM* that met general variant calling filters (described in the Methods section). Within the limits of long-read PacBio capacity for variant calling in highly amplified genomic regions such as the marked *clag2* CNV we report, we did not detect any mutations that could be examined. We agree that, given time and further selection, one or more of the many *clag2* copies could accrue mutations that further enable parasite growth, especially in light of ongoing evolution in *P. falciparum clag* genes (as discussed in the cited ref. 60).

*3. Figure 5C:re-check genomic copy number after tagging, as C3h-KO had 1 copy earlier and similar mRNA but the C3h-KO-Nluc has higher protein expression. CAS9 cleavage & repair at the locus could have led to changes in copy number.*

Figure 4B shows an amplicon in inverse PCR using *C3h-KO* template, suggesting that *C3h-KO* in fact has more than 1 copy of *clag2*; this presumably accounts for the higher protein content in *C3h-KO_C2NLuc_.* We opted not to pursue copy number determinations after transfection because: 1) individual transfection subclones will invariably have different *clag2* copy numbers, 2) our attempts to examine *clag2* conditional knockdown using the *glmS* riboswitch were unsuccessful, rendering precise determination of copy number unhelpful for understanding either transport or growth phenotypes, and 3) *clag2* copy number appears to vary dynamically in culture, as evidenced by selected increases or decreases upon transfer between RPMI and PGIM. To clarify this important point for readers, we have added “Cas9-mediated cleavage … all may alter *clag2* copy number.” p. 13, lines 7-9.

*4. Is CLAG2-dependent transport in C3h-KO-PGIM susceptible to inhibition by ISG-21 or residual transport inhibitors PRT1-4?*

We previously reported a modest change in ISG-21 affinity through dose-response studies with *C3h-KO* (PloS Path. 2020, ref. 18 of the revised manuscript). As this and other inhibitors from our various chemical screens are not specific for one or another inhibitor from various chemical screens we have performed, we felt this would be phenomenological and provide only limited insights that are outside of the scope of the present manuscript.

We thank the reviewer for bringing up PRT1-4, PSAC inhibitors that target a minor component of transport through the channel (termed “residual transport” because various observations suggest an alternate mechanism of solute recognition and permeation through PSAC). These compounds were relatively low affinity but had interesting growth inhibition phenotypes in combination with more traditional PSAC inhibitors (PMID: 26866812 published in 2016). Unfortunately, without the structure of the functional ion channel, these inhibitors were unable to substantively advance our understanding of nutrient permeation. We no longer have these screening hits, which were not pursued by our drug discovery pharma partners.

*5. Can the fact that CLAG3-loss has a major effect on growth in PGIM but lesser effects on transport and lysis be explained be differences in the intracellular concentrations of Ile required for growth vs lysis?*

The incomplete loss of PSAC-mediated transport in *C3h-KO* indeed contrasts with a more complete loss of parasite propagation in PGIM. In response to a similar question from another reviewer, we added “These findings are consistent … which remain unknown.” p. 7, lines 6-9.

Technically speaking, there is not a specific Ile concentration required for osmotic lysis. Osmotic lysis results more directly from the removal and replacement of impermeant extracellular solutes (specifically Na^+^ and its permeant counterion Cl^-^) by permeant organic solutes. The method is nonetheless useful because the kinetics of lysis are quantitatively related to Ile permeability, as we previously described (PMID: 12524269).

*Part III – Minor Issues:*

*Does synchrony and stage of C3h-KO alter the fraction of "dormant" parasites that drive recrudescence when switched back to RPMI?*

This question asks about S1 Fig., where we examined growth recovery of *C3h-KO* upon return to RPMI after variable durations of cultivation in PGIM. We did not attempt to synchronize and evaluate parasite stage in these stalled cultures because such studies were not possible with the small numbers of surviving parasites in PGIM.

*Figures would benefit from within-panel labels and have plenty of white space to fit them.*

We agree and have added within panel labels to Figures 4C, 5A and S2. All other figure panels have clear axis labels or obvious color-coding. We also clarified the conserved color-coding for the 4 parasites in all figures (KC5, *C3h-KO*, *KO-PGIM*, and *KO-rev* are black, red, cyan, and dark red respectively).

*FigS2A: repeating the primer diagram would be helpful for parsing the figure.*

We agree and have added ribbons for the WT and transfectant lines with primer position to this figure, now Fig. S3A.

*FigS2B: Is this the expected localization pattern for clag2? Is there any localization to the RBC membrane ?*

Yes, some export into RBC cytosol and membrane localization of CLAG2 is apparent in the revised Fig. S3B but, as with prior IFA studies from our and other groups (e.g. refs. 19, 24), a significant fraction of *clag* and *rhoph* protein is retained within the PVM. The best evidence for CLAG2 RBC membrane insertion is through protease susceptibility studies as we reported recently (see Fig. 2A of ref. 20).

*The discussion would be better off starting with the second paragraph as clag2 CNV is not an example of antigenic switching but rather a compensatory mechanism in response to the engineered loss of clag3 and selection with reduced nutrient serum, similar to other CNV that occur in response to drug selections.*

A similar point was raised by Reviewer #2, who stated “*Copy number variation was observed in an artificial parasite line with no functional clag3 gene …. Furthermore, large copy number variation has not been described in field isolates.*” Our findings actually suggest an alternate view, which challenges the prevailing dogma of rapid parasite adaptation being mediated exclusively by antigenic switching. The following observations support the comparison to antigenic switching: 1) Adaptation of *C3h-KO* to PGIM medium occurred in small-volume cultures within 2 months, with rare healthy parasites visible in smears quite early in the adaptation process. The kinetics of antigenic switching depend on the gene family and the specific parental paralog that is expressed before selection, but are somewhat faster. See Fig. 6G and Fig. 6H of PMID: 21620134 for antigenic switching rate for *clag3* genes, where half-maximal switching required 60-75 days (in both PSAC phenotype and RT-PCR experiments). 2) Although our selection did indeed use an engineered *clag3* knockout line, we point out that standard culture uses a supraphysiological medium (RPMI 1640 has very high concentrations of ~ all amino acids and vitamins, Table 1 of PMID: 23956690), growth at nonphysiologically low hematocrits, and typically depends on laboratory adapted lines. 3) There is strong evidence for copy number variations for *clags* between *Plasmodium spp.*, ranging from as few as 2 in *P. berghei* and *P. knowlesi* to over 35 copies in *P. blacklocki*. Even within a single species, *P. falciparum* isolates are also known to have variable *clag* copy number without engineered *clag3* knockout as ref. 60 identified clones having between 3 and 7 *clag* copies.

Based on our findings, it appears likely that changes in *clag* gene copy number may have happened many times in *Plasmodium* evolution in response to nutrient availability, either during entry into new host species or during marked changes in nutrient availability. With sufficient time, new *clag* copies could then accrue mutations that further refine channel properties such as selectivity for specific nutrients.

To clarify this point, we have made multiple revisions. See: “Our findings suggest that malaria parasites can change their DNA in response to sustained changes … into new host species.” p. 2, lines 8-10. “Although CNV generally cannot match … sustained changes in environment.” p. 14, lines 11-13. “When compared to other multigene copies, … range between 3 and 7 even without engineered *clag3* knockout [60].”, p. 15, line 22 – p. 16, line 4. “As with mutations and indels, … capacity for CNV under appropriate and sustained selective pressure.” p. 15, lines 15-21. We hope these changes fully address the question raised by this reviewer.

*Fig5C: Is the Nluc still active on the membrane even after being run on a denaturing gel? Or was this probed with anti-Nluc antibody? Non-experts in patch-clamping (like me) might benefit for a more context and elaboration of the results in Figure 3.*

Yes, NanoLuc is still active, yielding a luminescent signal on membranes after SDS-PAGE. This engineered luciferase is small, bright, and stable under conditions that denature most proteins. We have modified the Methods to include “using antibody-free blotting to measure luminescence on membranes [40].” p. 19, lines 19-20.

We agree that the Results section on patch-clamp measurement of Cl^-^ fluxes for KC5, *C3h-KO*, and *KO-PGIM* could use more context to help the broader community interpret the Results. We have added a new paragraph describing that uninfected cells have small whole-cell currents and the larger currents observed on infected cells reflect net Cl^-^ transport across the infected cell plasma membrane, and that the method is specific for PSAC. Please see “While uninfected human … Cl^-^ transport is abolished [24].” p. 9, line 19 – p. 10, line 2.

We also elaborated the Figure 3 legend with “Notice the larger … each clone’s channels.” and “voltage-dependence, apparent as steeper …, does not differ significantly between these clones.” p. 34, lines 8-10 and 15-16.
